# Honey Bee and Bumble Bee Antiviral Defense

**DOI:** 10.3390/v10080395

**Published:** 2018-07-27

**Authors:** Alexander J. McMenamin, Katie F. Daughenbaugh, Fenali Parekh, Marie C. Pizzorno, Michelle L. Flenniken

**Affiliations:** 1 Department of Plant Sciences and Plant Pathology, Bozeman, MT 59717, USA; kdaughenbaugh@gmail.com; 2 Department of Microbiology and Immunology, Bozeman, MT 59717, USA; alexander.mcmenamin@msu.montana.edu (A.J.M.); fenali10@gmail.com (F.P.); 3 Center for Pollinator Health, Montana State University, Bozeman, MT 59717, USA; 4 Biology Department, Bucknell University, Lewisburg, PA 17837, USA; pizzorno@bucknell.edu

**Keywords:** honey bee, virus, bumble bee, insect antiviral defense, RNAi, RNA-triggered antiviral defense, viral PAMP, dsRNA

## Abstract

Bees are important plant pollinators in both natural and agricultural ecosystems. Managed and wild bees have experienced high average annual colony losses, population declines, and local extinctions in many geographic regions. Multiple factors, including virus infections, impact bee health and longevity. The majority of bee-infecting viruses are positive-sense single-stranded RNA viruses. Bee-infecting viruses often cause asymptomatic infections but may also cause paralysis, deformity or death. The severity of infection is governed by bee host immune responses and influenced by additional biotic and abiotic factors. Herein, we highlight studies that have contributed to the current understanding of antiviral defense in bees, including the Western honey bee (*Apis mellifera*), the Eastern honey bee (*Apis cerana*) and bumble bee species (*Bombus *spp.). Bee antiviral defense mechanisms include RNA interference (RNAi), endocytosis, melanization, encapsulation, autophagy and conserved immune pathways including Jak/STAT (Janus kinase/signal transducer and activator of transcription), JNK (c-Jun N-terminal kinase), MAPK (mitogen-activated protein kinases) and the NF-κB mediated Toll and Imd (immune deficiency) pathways. Studies in Dipteran insects, including the model organism *Drosophila melanogaster *and pathogen-transmitting mosquitos, provide the framework for understanding bee antiviral defense. However, there are notable differences such as the more prominent role of a non-sequence specific, dsRNA-triggered, virus limiting response in honey bees and bumble bees. This virus-limiting response in bees is akin to pathways in a range of organisms including other invertebrates (i.e., oysters, shrimp and sand flies), as well as the mammalian interferon response. Current and future research aimed at elucidating bee antiviral defense mechanisms may lead to development of strategies that mitigate bee losses, while expanding our understanding of insect antiviral defense and the potential evolutionary relationship between sociality and immune function.

## 1. Introduction

### 1.1. Bees—Hymenopteran Insects That Play an Important Ecological Role as Plant Pollinators

There are over 4000 bee species in the order Hymenoptera, including those that are social or solitary, native or introduced, managed or wild [1]. Bees are important pollinators of plant species, including agricultural crops (e.g., almonds, apples, cherries, squash, tomatoes) and ecologically important plants. In the United States honey bee pollination is valued at 14.6 billion annually [2] and insect pollination worldwide is valued at $175 billion per year [3]. Due to their abundance and economic importance, most of the research on bee host—virus interactions has focused on honey bees.

Western honey bees (*Apis mellifera*) are eusocial, cavity nesting bees that are native to Europe, Africa and the Middle East; they were introduced into North America in the late 1600s [4,5,6]. The Eastern honey bee (*Apis cerana*) is a related but distinct species endemic to Asia and detected in Australia in 2007 [7,8]. Honey bee colonies consist of approximately 35,000 individual bees, including sterile female workers, a few hundred male bees (called drones) and a single reproductive female queen bee [9]. Honey bee colonies typically survive multiple years, while the longevity of individual worker bees depends on their caste (i.e., from six weeks to four months for worker bees, approximately eight weeks for drones and several years for queen bees [9]). The majority of the approximately 2.5 million honey bee colonies in the United States (US) are managed by professional beekeepers and are involved in pollinating the almond crop, which is the largest pollination event in the world [10,11]. Since 2006, US beekeeping operations have suffered approximately 33% annual losses, which is an increase from historic levels of approximately 12–15% [10,12,13,14,15,16,17]. Many biotic and abiotic factors contribute to these losses, including pathogenic infections, mite infestation levels, agrochemical-exposure, management, and lack of quality forage and habitat (reviewed in [18,19,20,21,22,23,24]). Viruses, including deformed wing virus (DWV), are one of the factors that contribute to individual bee and colony deaths.

Bumble bees, including *Bombus terrestris* and *Bombus impatiens*, are also important agricultural pollinators of crops such as tomatoes and peppers, as well as blueberries and other ecologically important plant species [25]. Bumble bees are ground nesting bees that live in small annual colonies with distinct solitary and social life cycle phases [26]. Unlike honey bees, bumble bees (*B. terrestris*) rear one generation per year [27]. This means that the queen survives one year and her reproductive daughters (gynes) start new colonies in the spring after an overwinter period of torpor (called diapause) [28,29]. There are numerous species of bumble bees, some of which have suffered high losses and local extinctions that are partially attributed to habitat destruction and fragmentation, chemical-exposure, pathogens, and climate change, [26,30,31,32,33,34,35,36,37]. The majority of bumble bee host pathogen research has focused on microsporidia (i.e., *Nosema bombi*) and trypanosomatid (i.e., *Crithidia bombi*) infections [38,39,40,41,42], though there is a growing body of virus literature, which is featured herein [43,44,45,46,47,48]. Recent metagenomic sequencing analysis of bumble bees (i.e., *Bombus terrestris*, *Bombus cryptarum*, and *Bombus pascuorum*) obtained from several locations in Belgium identified several known bee infecting viruses (e.g., black queen cell virus (BQCV), Varroa destructor virus-1 VDV-1/DWV-B, DWV), including potentially different strains as well as numerous new bee-associated viruses including (+)ssRNA, (−)ssRNA, and dsDNA viruses [49]. Future studies aimed at characterizing the full genome sequences, virion structure, potential pathogenicity, host-specific antiviral responses, and inter-taxa transmission of these viruses will greatly expand our understanding of bee virology [49]. Virus infections of social bees, including honey bees and bumble bees, may impact bee health at the superorganism (i.e., entire colony) and/or individual bee levels. Typically, colony population size is used as a proxy for colony health, whereas pathogen burden, life span, glandular protein content, and queen bee fecundity are used as proxies of individual bee health [12,14,16,50,51,52,53,54,55].

Solitary bees including alfalfa leaf cutter bees (*Megachile rotundata*), blue orchard or mason bees (*Osmia lignaria*), and many other native and wild bee species are important plant pollinators. Some are generalist pollinators, whereas others are specialist pollinators that primarily interact with one or just a few plants (reviewed in [56]). Interestingly, numerous studies indicate that agricultural systems that include both managed and native and wild bee species have improved crop yield [57,58,59,60]. Less is known about the health of these bees but in general habitat destruction, pathogenic infections, lack of quality forage, and agrochemical exposure are detrimental to bee health and population size (reviewed in [33]). Therefore, strategies that promote bee health including planting and/or maintaining pollinator forage, maintaining nesting sites (including bare earth for ground nesting bees), and reduced use of chemicals, particularly insecticides, will benefit all bee species. Though more research on the impact of viruses on solitary bees is needed, many studies have shown that these bee species are infected by viruses originally discovered in honey bees [61], as well as viruses and other parasites that may be unique to particular hosts [49]. For example, high throughput sequencing of the metatranscriptomes of eight wild bee species including five solitary bee species (i.e., *Andrena cineraria*, *Osmia bicornis*, *Osmia cornuta*, *Andrena fulva*, and *Andrena haemorrhoa*) in Belgium resulted in strong support for bee macula-like 2 virus infection of *A. haemorrhoa* and detection of several new partial virus genomes including a nege-like virus and a toti-like virus in *A. haemorrhoa *and *O. cornuta*, respectively [49].

A current focus of bee virus research is investigating intra- and inter- genera transmission of viruses. Though difficult to investigate, phylogenetic analyses of virus genome sequences obtained from co-foraging bee hosts have indicated that viruses are bidirectionally transmitted between managed and wild bee species [62,63,64,65] (reviewed in [66]). Additional studies are required to determine the extent of virus replication, as opposed to virus prevalence and pathogenesis across bee taxa. Inter-genera virus transmission is likely influenced by virus prevalence and abundance in bee populations, as well as the dynamic composition of bee and forb species in specific geographic regions. In addition, plant-pollinator networks and in turn pathogen transmission between co-foraging bees, are influenced by habitat loss and will likely be influenced by climate change [31,37]. Investigating the co-evolutionary history of specific virus-host pairs, host antiviral immune responses, and viral counter measures in numerous bee species will greatly enhance our understanding of bee virus ecology.

### 1.2. Bee Viruses

In this review we will use the term “bee virus”, though insect viruses generally have a broad host range and “bee viruses” can infect a variety of bee hosts, as well as ants and mites [67,68,69,70] (reviewed in [66]). Because of their role in agriculture, honey bees (*Apis mellifera*) are the most investigated bee species and thus the majority of bee-infecting viruses were discovered in honey bee samples. Most bee viruses are positive-sense single-stranded RNA viruses with approximately 30 nm diameter icosahedral capsids. These include Dicistroviruses (black queen cell virus (BQCV), Israeli acute paralysis virus (IAPV)), Iflaviruses (deformed wing virus (DWV), sacbrood virus (SBV), slow bee paralysis virus (SBPV)) and yet-to-be taxonomically classified viruses including chronic bee paralysis virus (CBPV) and the Lake Sinai virus (LSV) group (reviewed in [68,70,71]). Recent sequencing efforts have discovered new bee viruses from additional families (reviewed in [72]) including viruses with negative-sense RNA genomes and enveloped virions [73]. To date, only one bee-infecting DNA virus, Apis mellifera filamentous virus (AmFv), has been described [74]. For a more thorough review of bee virology, see Grozinger and Flenniken [75] and Chen and Siede [70].

Bee viruses are transmitted vertically within species and horizontally, both within species and between different bee genera [62,63,64,70]. Horizontal transmission is facilitated by food transfer (i.e., trophallaxis in social bees) between individual bees within a colony, and between colonies and bee species via the sharing of floral resources (i.e., nectar and pollen) [9,62,76]. Honey bee viruses are also transmitted within and between honey bee colonies by the ectoparasitic mite *Varroa destructor* (i.e., DWV, IAPV, KBV) [77,78,79,80,81,82,83,84,85]. Several studies suggest that DWV replication in mites and/or mite-mediated virus transmission impacts the diversity of viral genomes at both a geographic scale (i.e., mite induced bottleneck of DWV strains in the Hawaiian Islands [86]) and at the individual bee level [87,88]. Poor honey bee colony health is associated with high mite infestation coupled with DWV infection [83,85,89,90,91,92,93] and the seasonal dynamics of mite infestation and DWV abundance are strongly correlated [12,17,54,85,90,93,94,95]. The potential role of parasite-mediated virus transmission is under-explored for other bee species.

**Figure 1:**
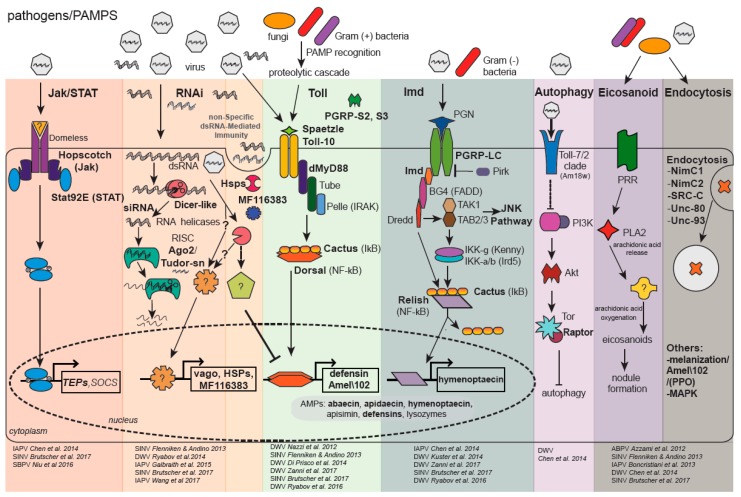
Honey Bee Immune Pathways—Highlighting Genes Implicated in Antiviral Immune Responses. The honey bee genome encodes major members of insect immune pathways including: Jak/STAT (Janus kinase/Signal Transducer and Activator of Transcription); RNAi (RNA interference); Toll via NF-κB (Nuclear Factor κB/Dorsal); Imd (Immune deficiency) via NF-κB/Relish; JNK (c-Jun N-terminal kinase); and MAPK (Mitogen-Activated Protein Kinases), as well as orthologues of genes involved in the heat shock response (Hsp), autophagy, eicosanoid biosynthesis, endocytosis, melanization, and prophenoloxidase (PPO) response. Bold text indicates genes and proteins differentially expressed in virus-infected honey bees and/or bumble bees. The first step in immune activation is host recognition of pathogen-associated molecular patterns (PAMPs) including viral dsRNA, bacterial peptidoglycans, and fungal β-glucans. In general, the Toll pathway is involved in defense against Gram(+) bacteria and fungi and the Imd pathway is activated by Gram(−) bacteria, but specific host-pathogen interactions are unique. This is particularly true for host—virus interactions since data from fruit-flies, mosquitoes, and honey bees indicate differential activation of immune genes and pathways. The Jak/STAT pathway is activated via ligand binding to the Domeless receptor; while *Drosophila melanogaster *(*Dm*) express Domeless ligands (*unpaired*, *upd*, *upd2*, and *upd3*), a honey bee *upd* orthologue has not been identified. Following Domeless-ligand binding, Hopscotch Janus kinases are transphosphorylated, leading to phosphorylation and dimerization of STAT92E-like proteins. Activated STATs transcriptionally regulate antimicrobial effectors TEP7 (Thioester-containing protein 7), TEPA, TEPB, and the Jak/STAT inhibitor SOCS (Suppressor of Cytokine Signaling). The honey bee genome also encodes for D-PIAS (Protein Inhibitor of Activated STAT), another inhibitor of the Jak/STAT pathway. The RNAi-pathway is initiated by *Dm*Dicer-2 cleavage of viral dsRNA into 21–22 bp siRNAs; *Am*Dicer-like shares ~30% aa identity with *Dm*Dicer-2. The siRNAs are then loaded into Ago2 (Argonaute-2), the catalytic component of the RISC (RNA Induced Silencing Complex). A single strand of the siRNA is retained in the RISC and used to specifically target cognate viral genome sequences for cleavage. In addition, *Dm*Dicer-2 serves as a dsRNA sensor that mediates a signal transduction cascade resulting in increased expression of *Dm*Vago, which suppresses viral replication. *Am*Dicer-like may serve as a dsRNA sensor, as honey bees have a *vago* orthologue which is up-regulated in DWV-infected honey bees, but not Sindbis-GFP-infected honey bees. In *B. terrestris*, Vago limits viral infection in fat bodies in a Dicer-dependent manner. Though the mechanism(s) of non-specific dsRNA-mediated antiviral responses in bees require additional characterization, a putative serine/threonine cyclin-dependent kinase (MF 116383) is involved in this virus-limiting response in honey bees. Additionally, several members of the heat shock protein family exhibit increased expression in Sindbis-GFP infected honey bees (i.e., *hsp90*, *activator of hsp90*, *60 kda hsp*, *10 kda hsp*, *hsp83-like*, *hsc70-4*, and *hsf5*), while dsRNA alone resulted in increased expression of *hsp90*. The Toll pathway is activated by a family of pathogen recognition receptors (PRRs) (e.g., peptidoglycan receptor proteins (PGRPs) and Gram(−) binding proteins) that bind fungal and bacterial PAMPs. The Toll pathway is activated in some insect host-virus combinations, although the activation mechanism is unknown. Following PAMP binding, a serine protease cascade results in cleavage of pro-Spaetzle into mature Spaetzle. The honey bee genome encodes two putative *spaetzle* orthologues, which bind the membrane-anchored Toll receptor. Toll dimerization results in the recruitment of dMyD88, Tube and Pelle. Pelle is likely involved in degradation of NF-κB inhibitors (e.g., Cactus-1, Cactus-2, Cactus-3), resulting in the release of transcription factors Dorsal-1A and Dorsal-1B. Nuclear translocation of Dorsal results in increased expression of antimicrobial peptides (AMPs) and *Amel\102*. The Imd pathway is activated by Peptidoglycan recognition protein LC (PGRP-LC) binding to diaminopimelic-containing peptidoglycan of Gram(−) bacteria, followed by activation of the adaptor protein Immune deficiency (Imd), Relish phosphorylation by the IKK complex (I_k_B kinase) and cleavage of Relish by the caspase Dredd (Death-related ced-3/Nedd2-like)_. _Relish transcriptionally regulates expression of AMPs and other genes involved in antimicrobial defense. The JNK pathway is also activated by TAK (Transforming growth factor-activated kinase 1) and TAB2/3 (TAK binding protein 2 and 3), resulting in AMP expression and/or apoptosis. In *Drosophila*, binding of vesicular stomatitis virus to the Toll-7 receptor promotes autophagy, likely by inhibiting the PI3/Akt/Tor (phosphatidylinositol 3-kinase/Protein kinase B/Target of rapamycin) pathway which suppresses autophagy. The honey bee genome encodes for one gene in the Toll-7/2 clade, *18-wheeler *(*Am18w*), which shares ~49% aa identity with *Dm*Toll-7 and ~45% aa identity with *Dm*Toll-2. The role of the Am18w protein in antiviral defense and autophagy in honey bees is unknown. In insects, Eicosanoid biosynthesis begins with the induction of PLA2 (Phospholipase 2) from signal cascades downstream of viral, fungal, or bacterial PAMP recognition. Activated PLA2 hydrolyzes arachidonic acid (AA) from cellular phospholipids. Eicosonoid production likely occurs via oxidation of AA by an unidentified enzyme. Eicosanoids are critical for nodulation and aid in phagocytosis, micro-aggregation, adhesion, and release of prophenoloxidase (PPO) from hemocytes. Experimental evidence also suggests endocytosis, melanization and MAPK pathways are involved in honey bee antiviral defense. Adapted with permission from Brutscher et al., Current Opinion in Insect Science, 2015 [102].

Virus infections in bees are primarily asymptomatic or they may result in deformity, paralysis, and/or death (reviewed in [70,89,96,97]) [98,99,100]. The extent of viral pathogenesis is influenced by biotic and abiotic stressors, including the synergistic negative effects of co-infection with multiple pathogens and/or agrochemical exposure, and governed by co-evolved host-virus interactions [37,101] (reviewed in [75]). The mechanisms of bee antiviral defense, which are described in greater detail below, include conserved immune pathways (i.e., Jak/STAT (Janus kinase/signal transducer and activator of transcription), JNK (c-Jun N-terminal kinase), MAPK (mitogen-activated protein kinases), NF-κB (i.e., Dorsal/Relish) mediated Toll and Imd pathways, RNA-trigged responses (i.e., RNA interference (RNAi) and a non-sequence-specific dsRNA mediated mechanism), autophagy, endocytosis, and melanization) (reviewed in [102,103]) (Figure 1).

### 1.3. Bee Virology

The epidemiology of bee viruses has not been thoroughly investigated, though there have been several insightful studies including the German Bee Monitoring Project [90], apiary level surveillance programs carried out by the US Bee Informed Partnership [12] and the Ministry of Agriculture in Spain [104], honey bee colony health and virus prevalence and abundance studies [12,13,14,15,16,17,53,54,55,97,105,106,107,108,109,110], and the ongoing Canadian National Honey Bee Health Survey [111]. These and other longitudinal monitoring studies have been instrumental in beginning to define how honey bee health relates to the prevalence and abundance of viruses, which varies by season, sampling date, and geographical location [54,105]. Several studies indicate that at particular times of the year weak colonies are associated with higher pathogen levels, including IAPV, LSV2, DWV, *Nosema ceranae*, and mites [12,13,14,15,16,53,54,55,90,95,97,106,109,110,112,113,114], though additional monitoring efforts are required to determine impacts of virus infection at the colony level.

To examine the impact of viruses on individual bees, Bailey, Ball, and others used semi-purified viruses from infected bees/pupae to catalogue the symptoms associated with particular viruses [70,100,115]. Symptomatic virus infections in honey bees include a “hairless” or “greasy” phenotype associated with CBPV, wing deformity and shortened or bloated abdomens caused by DWV, paralysis associated with IAPV and acute bee paralysis virus (ABPV) [116] and complications with larval development due to SBV and BQCV infections [70]. In addition, asymptomatic or covert infections may cause more subtle symptoms, such as the precocious foraging behavior and reduced lifespan associated with DWV-infections [117]. To date, only two infectious bee virus clones, BQCV [118] and DWV [119] have been described and only the DWV clone is currently available. Bee cell culture includes the use of primary cells obtained from embryos, larvae, and adults for short-term studies and a single immortalized cell line [120], which can be difficult to maintain [121]. Therefore, most bee virus research is carried out using viruses isolated from both naturally and experimentally infected bees and/or bee pupae [115]. Isolating viruses from naturally infected bees typically includes several co-purified viruses [115,121,122], though nearly pure virus isolates have been obtained (e.g., LSV2 [112]). Likewise, propagation of DWV and IAPV in pupae has resulted in relatively pure virus preparations [97,123,124,125]. The lack of infectious clones and robust cell culture models has made investigating bee viruses challenging but the use of model viruses, including Sindbis-GFP [126,127] and flock house virus (FHV) [128], and semi-purified virus inocula in individual bee and cultured cell experiments have provided insight into the mechanisms of antiviral defense [61,121,126,127,128,129].

The genomes of several bee species including *Apis mellifera *[130], *Apis cerana *[7], *Bombus terrestris*, *Bombus impatiens *[131], and *Megachile rotundata* have been sequenced and partially annotated [132]. Genomic information facilitates the use of molecular techniques (e.g., high through-put sequencing, qRT-PCR, cloning, and RNA interference-mediated gene knock-down) to investigate and understand bee host—virus interactions at both the colony and individual levels.

## 2. Bee Antiviral Defense

Bee antiviral defense mechanisms include dsRNA-triggered responses, hemocyte-mediated mechanisms (e.g., endocytosis, melanization and encapsulation), and conserved pathways including Jak/STAT, JNK, MAPK, and the NF-κB mediated Toll and Imd pathways (reviewed in [102,103]) (Figure 1).

Signal transduction cascades regulate the expression of genes, including those that are likely involved in limiting honey bee virus infections (reviewed in [102,130]), though very few studies have characterized the role of potential antiviral effector proteins in bees [85,126,128]. Gene function in bees is largely based on the roles of orthologous genes in model organisms, including *Drosophila melanogaster *[133,134,135,136,137,138,139] and mosquitos [140,141]. For example, *Drosophila melanogaster *encodes three NF-κB transcription factors (i.e., *relish*, *dif*, and *dorsal*) which are involved in promoting the expression of specific antimicrobial peptides (AMPs) [136,142]. Honey bees have two major NF-κB like transcription factors (i.e., *dorsal* and *relish*) involved in the Toll and Imd pathways, respectively. Honey bees lack *dif* but encode two dorsal homologues (i.e., *dorsal-1* and *dorsal-2*) and there are two isoforms of *dorsal-1* (i.e., 1A and 1B) [143]. Toll and Imd pathway activation increases expression of *defensin-1*, a Hymenoptera-specific AMP *hymenoptaecin* and both pathways govern expression of *abaecin *[143,144,145]. The roles of AMPs in host defense are best characterized in the context of bacterial and fungal infections where these cationic peptides act by disrupting pathogen cell membranes/walls [142,144,146]. The function of AMPs in virus infection is not yet known, though they serve as hallmarks of immune pathway activation [144,147,148].

There are several reports on the transcriptional level perturbations associated with virus infection and/or mite infestation of honey bees [87,93,97,125,126,127,149,150,151,152]. Even though these studies vary in terms of virus, infection route, inoculated versus field-acquired infections, bee age, sample date, and whether the presence of co-occurring infections were or were not examined, there were some general trends observed including increased expression of several AMPs (i.e., *apidaecin*, *hymenoptaecin*, *abaecin*, *lysozyme* and *defensins*), immune genes (e.g., *peptidoglycan recognition protein (PGRP)-S2*, *TEPs, prophenoloxidase, eater*), transcription factors, components of the RNAi machinery (i.e., *dicer *and *argonaute-2*) and differential expression of genes involved in metabolic pathways and cellular differentiation [87,93,97,125,126,127,149,151,152]. Several studies determined that *protein lethal*(*2*)*essential for life*-*like*, which encodes a protein in the small heat shock protein (Hsp20) family was differentially expressed in virus-infected bees [126]. The differential expression of this and other Hsp family proteins in virus-infected bees is intriguing, since the heat shock response is an important antiviral response in *Drosophila melanogaster* [153,154] and *Anopheles gambiae* [155].

Deformed wing virus infection of honey bees (*Apis mellifera*) is the most thoroughly examined virus-bee host pair [85,89]. Deformed wing virus infects both honey bee larvae and adults, and high DWV infection levels of larvae results in wing deformity in adulthood [85,87,117,156,157]. Investigation of DWV pathogenesis is complicated by the synergistic negative effects of both *Varroa destructor* mite parasitization and mite-mediated virus transmission, as well as impairment of the antiviral NF-κB/Dorsal-1A pathway as a result of exposure to neonicotinoid insecticides (i.e., clothianidin and imidacloprid) [101]. The complex interactions between host, virus, vector, neonicotinoid exposure and nutritional status, at both the individual bee and colony levels, were reviewed in this issue of *Viruses* by Nazzi and Pennacchio [158]. Together, several studies have demonstrated the key role of the NF-κB/Dorsal-1A pathway in limiting virus infection, including studies that determined that reduced expression of *dorsal-1A*, both experimentally and naturally, favored virus proliferation [93]. Furthermore, the NF-κB/Dorsal-1A pathway regulates the expression of *Amel\102*, which is an immune gene likely involved in melanization and encapsulation; *Amel\102* expression is reduced in DWV-infected bees [85,150]. Mite infestation and DWV levels are often correlated, though the immune response varies [87,93,150,159]. For example, when mite infested bees have low DWV levels the expression of genes in the Toll pathway are increased, whereas high DWV levels are associated with differential expression of genes in the JNK pathway [150]. In addition, RNAi is involved in controlling DWV infection, as evidenced by the production of viral siRNAs in DWV-infected bees [87,160] and increased cytopathology and DWV abundance in a persistently infected cell line after treatment with a potent RNAi inhibitor (cricket paralysis virus-1A protein) [121]. Furthermore and as described below, experimental introduction of DWV-targeting dsRNA reduced DWV abundance and DWV-associated mortality of both larvae and adult bees [87,161].

Nutritional status and metabolism also influence the outcome of virus infections in honey bees. Several transcriptome studies of virus-infected bees identified differential expression of genes involved in metabolic processes and bees fed higher quality diets had lower virus loads [97,125,126,150]. In addition, recent studies demonstrated that K_ATP_ channels, which respond to metabolic changes in the cell (e.g., the relative levels of ATP and ADP) play a role in limiting viral infection [128]. Specifically, honey bees fed a K_ATP_ agonist prior to challenge with FHV survived longer and had reduced virus levels compared to untreated bees or bees fed a K_ATP_ antagonist prior to virus challenge [128]. At least one study in *Drosophila melanogaster* links metabolic function to antiviral immunity by suggesting that K_ATP_ channels control FHV infection in an RNAi-dependent manner [162]. Future studies will determine if changes in metabolic function are a result of the hosts’ antiviral response or an energetic consequence of virus infections but these results provide a clear link between honey bee metabolism and antiviral defense. This is striking, given that naturally infected honey bees, even asymptomatic bees, routinely harbor over one billion virus genome equivalents per individual [105], covert DWV infections reduce longevity [117], and honey bee immune gene expression, which is associated with metabolism, varies with season [152].

### 2.1. Viral dsRNA-Triggered Antiviral Responses in Honey Bees and Bumble Bees, Including Sequence-Specific RNAi and Non-Sequence-Specific Pathways

During viral replication, most viruses produce long segments of dsRNA (e.g., replication intermediates of ssRNA viruses and RNA secondary structures in long transcripts of all viruses) that are recognized as PAMPs by host dsRNA-binding proteins. Host recognition of viral dsRNA may result in activation of sequence-specific RNAi and/or other antiviral immune pathways (e.g., Jak/STAT, Imd, and JNK) (reviewed in [102,134,135,141,163,164,165]).

### 2.2. Honey Bee Antiviral RNA Interference

RNAi is a post-transcriptional sequence-specific gene silencing mechanism that is involved in regulating gene expression in most organisms [166,167]. There are three distinct RNAi pathways including the microRNA (miRNA), piwi-interacting RNA (piRNA) and short-interfering RNA (siRNA) pathways (reviewed in [166,168]). The siRNA pathway is an important antiviral defense mechanism in plants, fungi, nematodes, and arthropods [140,166,168,169,170,171,172,173,174,175,176]. In brief, cytosolic virally-produced dsRNA is recognized and cleaved by an endonuclease enzyme Dicer (Dcr) into 21-nucleotide small interfering RNAs (siRNAs). The siRNAs are then loaded into the RNA-induced silencing complex (RISC) which contains the protein Argonaute-2 (Ago2). Once loaded into RISC, one of the siRNA strands remains associated with Ago2 (i.e., the guide strand), while the passenger strand is degraded by Ago2 and the endonuclease C3PO (component 3 promoter of RISC) [177]. The guide-strand binds to a complementary target single-stranded RNA, leading to Ago2-mediated cleavage of the target RNA (reviewed in [166]). The antiviral role of RNAi was first reported in solitary insects including *Drosophila melanogaster* infected with FHV, which is a positive-sense single stranded RNA (ssRNA) virus [178]. Later, flies deficient in *ago-2* were shown to be hypersensitive to viral infection [179], further implicating particular members of the RNAi pathway in antiviral defense. Interestingly, the systemic spread of a sequence-specific RNAi response to uninfected cells, mediated by a dsRNA uptake pathway, was shown to be necessary for an effective antiviral immune response [180,181]. Recent* Drosophila* studies have revealed that hemocytes and transposon-encoded reverse transcriptases are involved in amplification and systemic spread of siRNA-mediated antiviral defense [182,183,184].

The role of RNAi in honey bee (*Apis mellifera*) antiviral defense was demonstrated in laboratory-based experiments in which bees and/or larvae that were fed virus-specific dsRNA harbored reduced levels of virus, as compared to controls [127,161,185,186]. Adult bees fed IAPV-specific dsRNAs had less mortality [186] and lower virus loads [97] post IAPV-infection as compared to bees fed non-sequence specific dsRNA. Likewise, larvae that were fed DWV-specific dsRNA prior to inoculation with DWV had a reduced percentage of wing-deformity and lower DWV virus load than larvae fed non-sequence specific dsRNA (i.e., dsRNA-GFP), although survival was not impacted [161]. The same study determined that adult bees fed dsRNA-DWV prior to DWV inoculation had reduced viral loads and increased longevity, compared to DWV-infected bees [161]. Intriguingly, particularly in the context of later studies described below, adult bees fed dsRNA-GFP prior to DWV inoculation also had increased longevity compared to DWV-infected bees but the relative abundance of DWV RNA equivalents was similar to DWV-infected bees that were not fed dsRNA [161]. Together, these laboratory-based studies indicated that siRNAs and/or dsRNAs may be useful antiviral treatments, though other studies indicated dsRNA had additional biological impacts on bees [44,47,126,187,188,189,190]. A field study in which honey bee colonies were fed both IAPV-specific dsRNA (Remebee-IAPV^®^) and IAPV determined that dsRNA-treated colonies in one of two locations had larger adult bee populations and produced a greater amount of honey, than colonies that were only fed IAPV, though virus abundance was not assessed [191]. A more recent, colony level study characterized the siRNAs (21–22 nt) produced in bees obtained from naturally and experimentally IAPV-infected colonies [160]. Interestingly, the majority of sequenced IAPV-siRNAs corresponded to the negative sense-strand, whereas the viral siRNAs corresponding to DWV matched both strands [160]. Bee samples from both Colony Collapse Disorder (CCD)-affected and unaffected colonies contained viral siRNAs, indicating that even in the context of CCD, honey bees mounted an RNAi-mediated antiviral response [160]. 

Further support that RNAi plays an important antiviral role in honey bees is that the expression of *dicer-like* and *ago-2* is higher in virus-infected bees (i.e., IAPV or a model virus Sindbis-GFP), as compared to mock-infected bees [125,126]. However, increased expression of the RNAi machinery was not observed in DWV-infected honey bees [87]. In addition, RNAi-mediated reduction of honey bee gene expression has become a useful tool to investigate gene function at different honey bee developmental stages including embryos [192], larvae [193,194], pupae [143,195], and adult bees [101,126,196,197,198,199] and provides further evidence that honey bee RNAi machinery is functional.

### 2.3. Honey Bee Non-Sequence-Specific dsRNA-Mediated Antiviral Response

While sequence-specific RNAi is an important honey bee antiviral defense mechanism and experimental gene silencing tool, dsRNA also triggers a general non-sequence-specific antiviral response (reviewed in [126,172]). Initial observations that dsRNA treatment altered honey bee gene expression in a sequence independent manner were made in the context of targeted gene knock-down studies [187,190]. As described previously, dsRNA is a viral PAMP that is likely recognized by dsRNA recognition proteins, which in turn activate signal transduction cascades that result in an antiviral transcriptional profile [126,127]. The first study that demonstrated a non-specific dsRNA-mediated virus-limiting response in honey bees came from laboratory-based experiments in which adult honey bees were co-injected with Sindbis-GFP and virus-sequence specific dsRNA, non-sequence specific dsRNA, or poly(I:C) (a synthetic analogue of dsRNA), all of which reduced viral abundance at 72 h post infection [127]. Perturbations in honey bee gene expression in response to either virus or dsRNA treatment at 72 h post-inoculation were identified via microarray analyses compared to mock-infected control [127]. A more comprehensive examination of dsRNA-triggered honey bee antiviral defense mechanisms utilized transcriptome sequencing (RNASeq) to identify differentially expressed genes (DEGs) at 24, 48, and 72 h post virus-infection and/or dsRNA administration [126]. This study determined that the number of DEGs increased over the course of virus infection and that many genes had unique temporal dynamics. Honey bee genes that were differentially expressed in virus-infected and/or dsRNA treated bees included *hopscotch* in the Jak/STAT pathway, *toll-10* and *tube* in the Toll pathway, *pirk* and *jra* in the Imd pathway, antimicrobial peptides (i.e., *apidaecin1*, *hymenoptaecin*, *abaecin* and *defensin*), numerous heat shock response genes, *dicer* and *ago-2* and *scavenger receptor class c*, which is involved in dsRNA uptake in *Drosophila*; most DEGs were functionally uncharacterized [126]. The virus-limiting functions of two of the identified honey bee antiviral genes (i.e., *dicer*, NCBI GeneID 552127) and a *probable cyclin-dependent serine/threonine kinase (*NCBI GenBank MF116383) was confirmed in individual bee gene knock-down experiments. Reduced expression of either *dicer* or *MF116383* resulted in greater virus abundance [126]. In *Drosophila melanogaster* Dicer is involved in both specific and non-specific dsRNA responses including activating expression of the antiviral gene *vago* [200], though the mechanism of Vago-mediated virus reduction in *Drosophila* is not yet known. In mosquitos, Vago is a secreted protein that limits virus infection by linking the siRNA and Jak/STAT pathways through an unknown receptor [135,201,202]. *Vago *expression is increased in DWV-infected honey bees [87], though the neither the signal transduction pathway or transcription factor(s) involved have been identified. Furthermore, *vago* is not universally up-regulated in response to virus infection in honey bees (e.g., SINV-GFP, IAPV) [125,126].

Recognition of non-self virally-produced dsRNA is the first step in activation of an antiviral state in many organisms. Cytosolically-located dsRNA recognition proteins include DExD (Asp-Glu-x-Asp) box helicases, such as Dicer, Retinoic acid-inducible gene I (RIG-I), and Melanoma differentiation-associated protein-5 (MDA-5), Protein kinase R, and endosomal Toll-like receptor 3 in diverse organisms including invertebrates and mammals [44,47,200,201,202,203,204,205,206]. Though identification of the honey bee proteins that detect and respond to dsRNA is ongoing, we hypothesize that further investigation of this non-sequence specific dsRNA virus limiting response in honey bees will result in the identification of genes that will have conserved antiviral roles in other invertebrate and vertebrate organisms.

### 2.4. Bumble Bee RNA Interference

Bumble bee and honey bee lineages diverged approximately 90 million years ago [207]. Similar to honey bees, the bumble bee (*B. terrestris*) RNAi machinery (i.e., *ago-2*, *dicer-2*) exhibits slightly increased expression in the context of virus infection (i.e., IAPV and SBPV) [44,189]. Increased expression of key RNAi genes likely explains the observed enhancement of RNAi-mediated host gene knock-down in IAPV-infected *B. terrestris* [189]. The RNAi response is not very effective at reducing IAPV-abundance, as levels in experimentally inoculated bumble bees increased by over 1000-fold in spite of activation of RNAi [189]. This result could be partially attributed to tissue specific variation of RNAi efficacy [44,208,209]. However, even experimental reduction of *dicer-2* expression in *B. terrestris* did not result in increased abundance of IAPV or SBPV [44]. IAPV, which is transmitted orally among bumble bees, was injected in these studies and injection of IAPV results in a more virulent infection than oral inoculation [44,189], therefore additional studies are required to determine if the route of virus-inoculation impacts host-pathogen interactions and the efficacy of systemic RNAi. To further examine the virus-limiting role of RNAi, bumble bees were infected with cricket paralysis virus (CrPV), which is a model virus that encodes a viral suppressor of RNAi (VSR) [210] and as expected, the RNAi response was reduced [189]. This study did not find evidence of IAPV-mediated suppression of RNAi and the putative IAPV-1A-like VSR protein [97] was not detected by mass spectrometry analysis of infected bee samples [189].

### 2.5. Bumble Bee Non-Sequence-Specific dsRNA-Mediated Antiviral Response

Similar to honey bees, viral dsRNA triggers both sequence-specific RNAi and non-sequence specific virus limiting responses in bumble bees, though the non-sequence specific pathway may be even more important for bumble bees [44,47,189]. This was demonstrated by a study in which bumble bees (*Bombus terrestris*) were fed IAPV only, or IAPV and either virus-sequence specific dsRNA or non-sequence-specific dsRNA (ns-dsRNA-GFP, 455 bp) for six consecutive days, beginning three days prior to virus-inoculation [47]. Survival was monitored for 22-days post-infection and revealed that bumble bees treated with non-sequence specific dsRNA better survived IAPV infection (60%) than bumble bees treated with virus-specific dsRNA (10%). However, in parallel, independent experiments that examined the potential effect of IAPV-dsRNA length (293 bp, 443 bp and 586-bp) in addition to sequence specificity, virus abundance in bumble bee heads was reduced in all dsRNA-treated and virus-infected bees, as compared to bees that were virus-infected and not treated with dsRNA [47].

In addition to dsRNA-mediated antiviral responses, there are other immune pathways that likely play important roles in antiviral defense in bumble bees. Targeted analysis of canonical insect immune gene expression in virus-infected bumble bees unexpectedly indicated that *BtSVC-vago* (which is also referred to as Single von Willebrand factor C-domain protein (SVC) vago) expression was reduced in IAPV-infected bees and not affected in SBPV-infected bees, unlike the observed up-regulation of this important antiviral gene in *Drosophila melanogaster* [44]. Experimental reduction of *BtSVC-vago *expression did not impact IAPV or SBPV abundance in the abdomen [47], however additional tissue specific analyses determined that *BtSVC-vago* reduction resulted in greater IAPV abundance in the fat body [211]. Similar to regulation of *Dmvago *by *Dm*Dicer*-2*, *BtSVC-vago *expression is governed by *Bt*Dicer-2 [211]. Reduction of *BtSVC-vago* expression did not impact *Btdicer-2 or Bthop* levels in fat bodies and thus to date a connection between *Bt*SVC-vago expression and Jak/STAT activation has not been established [211]. However, reduced expression of *Bthop *resulted in higher SBPV load 48 h post-infection, implicating the Jak/STAT pathway in bumble bee antiviral defense [44] (reviewed in [102]) (Figure 1). To date, the mechanism by which down-regulation of *BtSVC-vago* regulates expression of four AMPs (*Btabaecin, Btapidaecin, Btdefensin *and *Bthymenoptaecin*) remains to be elucidated [211].

## 3. Conclusions

RNA virus infection results in the production of dsRNA molecules (e.g., virus replicative intermediates, RNA secondary structure, dsRNA genomes) in host cells. This viral pathogen PAMP is recognized as non-self by PRRs across diverse taxa, including plants, invertebrates (e.g., oysters, shrimp, nematodes, ticks, fruit flies, sand flies, mosquitos, wasps, and bees) and mammals [176,212,213,214,215,216,217,218,219,220]. Recognition of dsRNA by host PRRs induces distinctive antiviral responses across different hosts. These include virus-specific RNAi in plants, nematodes, and arthropods and non-sequence specific dsRNA-mediated induction of pathways that result in an “antiviral state” that limits virus replication (e.g., mammalian interferon response, *C. gigas* oyster type I interferon-like response, *A. mellifera* and *B. terrestris *virus-limiting responses*)*. The extent to which these pathways are involved varies for each co-evolved virus—host pair. Overall the role of RNAi is greater in plants, fruit-flies, and mosquitos, while the role of other immune pathways including Jak/STAT, JNK, Toll, and non-sequence specific dsRNA-triggered pathways are more important in bees, oysters, and mammals. Importantly, these generalities are only true for the specific species that have been studied including *Arabidopsis thaliana*, *Xanthomonas oryzae*, *D. melanogaster*, *A. gambiae, A. mellifera*, *B. terrestris*, *C. gigas*, *Mus musculus*, *Homo sapiens*, and others.

The antiviral response(s) that are most important for any particular host-virus pair cannot be assumed based on broad organismal classification (e.g., insects, invertebrates, mammals)—each must be empirically determined. Since all host-virus interactions are inexorably complicated by the history of conflict and evolution shared (or not shared in the case of model viruses) by the host and a virus. These unique histories are reflected in the differential transcriptional responses and extent of parallel activation and/or cross-talk between host immune pathways, as well as identification of the virus-evolved counter defense mechanisms, including suppressors of RNAi and other immune pathways [140,221].

The parallels that exist between the antiviral responses, including the general, non-sequence specific dsRNA-triggered induction of an antiviral state, in organisms separated by large evolutionary distances including honey bees, bumble bees, sand flies, shrimp, oysters, and mammals, are very intriguing. Furthermore, it is interesting to hypothesize that in social bees this response may have evolved to rapidly respond to viruses and limit their transmission in the crowded hive environment and in the context of behaviors (e.g., trophallaxis) that promote virus transmission between individuals within the super-organism. Investigation of antiviral defense in some of the thousands of under-explored bee species, will further our understanding of the general and specific mechanisms that bees have evolved to combat specific viruses. This short review highlights studies that have contributed to our current understanding of bee antiviral defense mechanisms. Under-explored, burgeoning research areas include elucidation of the roles of alternative splicing [127,222], epigenetic regulation [125,223], and transgenerational immune priming in bee antiviral defense [224,225]. Further examination of antiviral RNAi, including immune memory as a consequence of RNA virus integration into the bee genome [226] and potential transposon-mediated amplification of virus-targeting secondary RNAs (as described in *Drosophila melanogaster *[182,183,184]) is also important. Lessons learned from evolutionary distant organisms, including those described in this special issue of *Viruses* focused on “Antiviral Defense in Invertebrates”, may help guide these studies.
